# New Appliances and Things Medical

**Published:** 1899-10-21

**Authors:** 


### NEW APPLIANCES AND THINGS MEDICAL.

### NEW MANTLELESS INCANDESCENT GAS BURNER.

(Billing's Burner Syndicate, 180, Wardour Street, London, W.)

This new burner, which is invented by Mr. Eardley Billing, is simple in construction, and inexpensive to instal, but according to the generally accepted meaning of the word "incandescent" it seems unfortunately named. The term incandescent as applied to a gas light is now generally recognised as meaning that the light is due not to the gas itself, but to something rendered incandescent by the heat produced in its combustion.

